# Changing places to study short-term effects of air pollution on cardiovascular health: a panel study

**DOI:** 10.1186/s12940-018-0425-7

**Published:** 2018-11-19

**Authors:** Hans Scheers, Tim S. Nawrot, Benoit Nemery, Lidia Casas

**Affiliations:** 1 Environment and Health Unit, Department of Public Health and Primary Care, KU Leuven, Herestraat 49, O&N I, PB 706, 3000 Leuven, Belgium; 2 Centre for Environmental Sciences, UHasselt, Diepenbeek, Belgium

**Keywords:** Air pollution, Carotid stiffness, Blood pressure, Quasi-experimental study

## Abstract

**Background:**

Short-term exposure to ambient air pollution triggers acute cardiovascular events. Here, we evaluate the association of exposure to ambient air pollution with two intermediate cardiovascular endpoints: blood pressure and carotid stiffness.

**Methods:**

In a one-year panel study, we included 20 healthy volunteers (10 male-female couples aged 59–75 years) with air pollution and health parameters measured every two months at their region of residence (Leuven, Belgium) and twice during two ten-day periods in two locations, one with higher (Milan, Italy) and one with lower (Vindeln, Sweden) air pollution levels (220 observations). We measured blood pressure, carotid arterial stiffness, personal exposure to NO_2_, and ambient concentrations of PM_10_, PM_2.5_, and NO_2_. We used linear mixed models to evaluate the associations between the health outcomes and the air pollutants.

**Results:**

Compared with Leuven, exposure to pollutants was higher in Milan and lower in Vindeln, with the highest contrast for NO_2_ (median 20.7 μg/m^3^ (IQR:7.4) vs 65.1 μg/m^3^ (9.0) and 4.5 mg/m^3^ (0.8), respectively). We did not observe significant associations between either systolic or diastolic blood pressure and variations in air pollution. However, we found significant associations between arterial stiffness and 5 day average exposure to the studied pollutants. The strongest associations were observed for PM_10_ with carotid distensibility (DC) and compliance (CC) coefficients, and the young elastic modulus (YEM): 4.3% (95%CI:7.0;1.5) increase in DC, 4.7% (95%CI:7.1;2.3) increase in CC and 4.2% (95%CI:1.1;7.3) decrease in YEM for each 10 μg/m^3^ decreases in PM_10_.

**Conclusions:**

Our study suggests that short-term exposure to air pollution results in reductions in carotid elasticity among elderly population.

**Electronic supplementary material:**

The online version of this article (10.1186/s12940-018-0425-7) contains supplementary material, which is available to authorized users.

## Introduction

Ambient air pollution is an important cause of respiratory and cardiovascular morbidity and mortality [1, 2]. Overall, 3.7 million deaths and 3.1% of disability-adjusted life years (DALY) worldwide are attributed to air pollution, placing it in the top 10 of risk factors [3]. It has been abundantly demonstrated that short-term exposure to air pollution (hours to a few days of exposure) can trigger acute events such as myocardial infarctions [4–6], whereas long-term exposure (after several years of exposure) has been linked to both the onset of acute events and the development of chronic diseases [7, 8]. In addition to epidemiological research, controlled-exposure studies in animals and humans have provided insight into possible physiological pathways underlying the relationship between inhalation of pollutants and cardiovascular and respiratory health. These pathways have been reviewed recently [9–11].

In this study, we combine the advantages of epidemiological and experimental studies to investigate the associations between air pollution and intermediate cardiovascular health endpoints. To achieve our objective, we deliberately moved a panel of study volunteers from their living area around Leuven, Belgium [yearly average PM_10_ (particulate matter) around 30 μg/m^3^] for several days to locations with contrasting levels of air pollution: Milan, Italy (> 50 μg/m^3^) and Vindeln, Sweden (< 10 μg/m^3^), i.e. locations representative for the highest and lowest levels of PM pollution in Europe, respectively [12–14]. We quantified several health-related endpoints that have been identified as intermediate steps between exposure and disease [15–18]. Here, we present the results for the association between air pollution concentrations and blood pressure and carotid stiffness, as intermediate endpoints in the associations between air pollution and cardiovascular diseases.

## Methods

### Study design and participants

We conducted a one year panel study in healthy elderly volunteers and measured multiple health endpoints and exposure to air pollution in locations with differing ambient air pollution levels. We included 10 healthy retired male-female couples with both partners fulfilling the following inclusion criteria: age approximately 60–75 years, never or > 1 year former smokers, good general health, willing and available to travel during the study period, and fluent in Dutch. We excluded persons with mobility problems; a history of cardiovascular disease (except uncomplicated hypertension), cancer, or other diseases that could interfere with the measurements or would represent a risk during travel. All participants were given detailed oral and written information on the study and gave written informed consent. The study was approved by the Ethical Committee of KU Leuven (S55482).

**Fig. 1:**
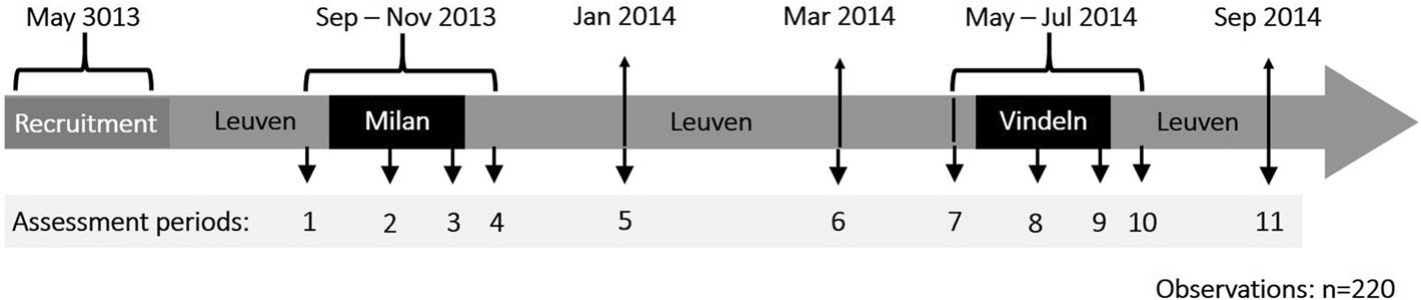
Timeline of the study. All variables mentioned in the text were measured in 20 study volunteers in all 11 periods, except for and plasma levels of cholesterol and glucose which was only measured at baseline (assessment period 1)

From September 2013 to September 2014, we collected data over 11 measurement time points: seven in Leuven (Belgium), two during a 10-day stay in Milan (trip days 5 or 6, and 9 or 10), and two during a 10-day stay in Vindeln (trip days 5 or 6 and 9 or 10), a rural area near Umeå, northern Sweden (Fig. 1). To limit differences in temperature between the two trips, we stayed in Milan in October (2013, average daily temperature 13 °C) and in Vindeln in June (2014, average daily temperature 14 °C) [19]. Environmental data form monitoring stations and personal exposure samplers was collected during each measurement time point and on the four days prior to it. Clinical measurements were performed in adequate study rooms at the Universitair Ziekenhuis Leuven, the Ospedale Maggiore in Milan, and Umeå University. We collected blood, sputum and urine samples, measured blood pressure, endothelial function and carotid stiffness, the study volunteers underwent cognitive tests and spirometries, their physical activity was measured with accelerometers, and they were administered health questionnaires and diet diaries. To further describe the baseline health status of our study participants we determined (at baseline) plasma levels of cholesterol and glucose in fasted blood samples. Additional information on the study design and population is provided elsewhere [20]. The study presented here focuses on the results for blood pressure and carotid stiffness measurements.

### Cardiovascular intermediate endpoints: Blood pressure and carotid stiffness

Systolic (SBP) and diastolic blood pressure (DBP) were measured according to guidelines of the European Society of Hypertension [21], with an automated device (Stabilograph, Stolberg, Germany). After the subject had rested for at least 10 min, blood pressure was measured five times consecutively in sitting position. We used the average of the last two measurements for analyses, and we calculated pulse pressure (ΔP=SBP–DBP), and mean arterial pressure as DBP + ΔP/3.

Carotid stiffness was measured by using an ultrasound device with automatic boundary detection software in RF-mode (MyLabOne, Esaote Benelux, Maastricht, The Netherlands) according to previously reported protocols [22]. Participants rested for 10 min in a supine position before starting the measurements. All measurements were performed by the same trained investigator by longitudinal scanning of a 1 cm segment of the right common carotid artery at 1 cm proximally to the dilatation of the carotid bulb visualizing the lumen-intima and media-adventitia interfaces of the far arterial wall. Carotid intima-media thickness was determined under three different angles (90°, 130° and 180°).

We averaged diastolic artery diameter and systolic increase in diameter over three consecutive ultrasound measurements, each spanning eight cardiac cycles. We used D and ΔD to calculate four parameters related to arterial stiffness [23, 24]. Carotid distensibility (DC) and compliance (CC) coefficients are inversely related to arterial stiffness, and pulse wave velocity (PWV) is a direct measure of arterial stiffness. Young’s Elastic Modulus (YEM) combines measures of arterial wall elasticity with intima media thickness. Thus, we expected to observe direct associations with air pollution for PWV and YEM and inverse associations for DC and CC. Intra-observer coefficients of variation ranged from 5.2 to 10.1% for the different stiffness parameters, indicating good reproducibility of measurements [17].

### Air pollutants

Participants lived within a maximum distance between the residences of 45 km. We estimated their daily average residential exposure to PM_10_, PM_2.5_, and NO_2_ using interpolated values in 4 by 4 km grids, based on the Belgian telemetric air quality network [25]. In Milan, daily averages from eight monitoring stations in the city were calculated using the information provided by the online database of the Regional Agency for the Protection of the Environment in Lombardy (ARPA Lombardia: http://www.arpalombardia.it) [26]. In Vindeln, we averaged daily data from the nearest monitoring stations in Umeå, Skellefteå and Strömsund. In addition, we measured personal exposure to NO_2_ using Radiello diffusive samplers (Sigma-Aldrich, Bellefonte, PA, USA). Depending on the measurement period, six to 19 participants wore the clip-on device during five days prior to each health assessment day in Leuven or to the last health assessment days in Milan and Vindeln. The number of samplers distributed was lower during the trips because all participants stayed at the same hotel and performed group activities during the day. In Belgium, one sampler was provided to each couple. However, to check potential differences in exposure within the couples, we provided 19 samplers in one measurement period. No significant differences were observed within couples. Personal exposure to NO_2_ was expressed as average concentrations (μg/m^3^) [27]. The quantification of average exposure to NO_2_ was performed at the lab of the Fondazione Salvatore Maugeri (Padova, Italy).

### Potential confounders

In this study, we included information obtained by face-to-face interviews on smoking status (never or former), medication use for hypertension, and having a cold. Regarding smoking status, our study included 10 former smokers who had stopped smoking 6 to 43 years (average = 29 years) previous to the start of the study and who smoked 0.6 to 18 packs-year (average = 6.5). In addition, we included heart rate measured with electrocardiogram, and considered physical activity (number of steps measured with an accelerometer) performed during the week previous to the cardiovascular measurements, and alcohol consumption reported on a diet diary for seven days prior to the measurements.

Environmental potential confounders such as daily temperature and relative humidity during the study period were obtained from local meteorological websites for Belgium (http://www.meteobelgie.be) and Milan (www.ilmeteo.it) and an international website for Umeå (http://www.wunderground.com).

### Statistical analysis

Statistical analyses were performed in SAS 9.4 (SAS Institute, Cary, NC, USA). We investigated associations between health parameters and exposure to air pollution by using linear mixed models with variance components covariance structure, accounting for the repeated-measures design of the study. ‘Acute’ effects of air pollution were estimated as 5 day effects by calculating the average of lag days 0 to 4 (referred to as ‘av04’), where lag 0 is the daily average of pollutant concentrations on the day of the health measurement, lag 1 the day before, lag 2 two days before, lag 3 three days before and lag 4 four days before. Thus, we obtained an average of daily averages of 5 consecutive days including the day of the health measurement and the 4 days prior to them. Personal exposure to NO_2_ was available as five day average concentrations [27] and included measurements of 4 days prior to the health measurements and of the day of the health measurements but only until the hour of its performance. During the trips, personal exposure to NO2 was measured only once (during 5 days). In the mixed models we used all repeatedly measured cardiovascular intermediate endpoints and the NO_2_ exposure, which reflects the average exposure during the trip.

The models were adjusted for age at baseline, sex, date of measurement, ambient temperature, relative humidity, heart rate, having a cold, high blood pressure medication, and smoking status. The models for the carotid stiffness measurements were additionally adjusted for mean arterial pressure. The inclusion of information on physical activity and alcohol consumption in the models did not modify the effect estimates and was, therefore, excluded in the final models. We tested the assumption of normal distribution of the error terms by visual inspection of the Q-Q plots of residuals. For PWV, DC, CC, and YEM, this assumption was only met after log10-transformation. Therefore, results for these outcomes are presented as % change and their 95% confidence intervals (CI), whereas parameter estimates of all other analyses are unit changes (beta coefficients) and their CI.

Travelling results in temporary changes in lifestyle as compared with lifestyle patterns followed at home. For example, it may involve changes in physical activity, time spent outdoors, diet, etc. To explore the potential effect of the trips on our results, we performed sensitivity analyses including only the measurements taken in Belgium. In addition, we excluded individuals taking antihypertensive medication because this may attenuate the response to air pollution, and we performed 3-level models including individual and couple as random effects to rule out a potential effect of correlated exposures and risk factors within the couple. Finally, we tested potential effect modification of sex and age by including interactions between the pollutants and these variables in the models.

## Results

All 20 participants completed the study in September 2014, without any dropout or missed measurement time point. Table 1 summarizes the main characteristics of the study population at baseline. Six participants took high blood pressure medication during the whole study period, one male started taking this medication after period L2 (Fig. 1). Five-day average concentrations of PM_10_, PM_2.5_, and NO_2_ are presented in Table 2. Personal exposure to NO_2_ was clearly highest in Milan (65 μg/m^3^) and lowest in Vindeln (5 μg/m^3^), with intermediate values for Leuven (21 μg/m^3^). Average concentrations of PM_10_, PM_2.5_ and NO_2_ from monitoring stations were highest in Milan, but no big differences were observed between Leuven and Vindeln.

**Table 1:** Baseline characteristics of the study participants (*n* = 20)

	Median (range)
Age, y	65 (58–76)
Sex (female)^a^	10 (50%)
Body-mass index, kg/m^2^	24.3 (18.9–29.4)
Smoking status^a^	
Former	10 (50%)
Never	10 (50%)
Plasma cholesterol, mg/dL	
Total	206 (144–282)
LDL	133 (57–212)
Plasma glucose, mg/dL	99 (86–131)
Medication for hypertension^a^	6 (30%)
Blood pressure, mm Hg	
Systolic	132 (109–165)
Diastolic	80 (65–105)
Pulse pressure	49 (40–68)
Carotid stiffness	
PWV, m/s	8.64 (6.65–20.16)
DC, 10^− 3^/kPa	0.01 (0.00–0.02)
CC, mm^2^/kPa	0.66 (0.04–1.66)
YEM, kPa	0.77 (0.48–2.10)

^a^n and %

*PWV* Pulse wave velocity, *DC* Distensibility coefficient, *CC* Compliance coefficient, *YEM* Young’s elastic modulus

**Table 2:** Description (mean and standard deviation) of five day average concentrations (μg/m^3^) of PM_10_, PM_2.5_ in each measurement time point performed in Leuven (Belgium), Milan (Italy) and Vindeln (Sweden) between September 2013 and September 2014

				Monitoring stations			Personal samplers
Year	Month	Location	Visit name	PM_10_	PM_2.5_	NO_2_	NO_2_
2013	September	Leuven	L1	21.9 (4.6)	15.9 (5.6)	25.1 (6.8)	29.8 (5.3)
	October	Milan	M1	35.5 (2.6)	31.5 (2.6)	54.9 (1.6)	–
			M2	28.9 (2.1)	19.1 (1.5)	46.3 (0.9)	63.7 (4.7)
	November	Leuven	L2	9.4 (0.8)	5.9 (0.8)	19.4 (4.2)	28.5 (8.9)
2014	January	Leuven	L3	15.0 (2.5)	11.4 (2.2)	26.0 (5.2)	21.5 (8.1)
	March	Leuven	L4	19.8 (4.7)	14.5 (5.0)	27.5 (8.2)	21.4 (6.0)
	May	Leuven	L5	13.9 (2.0)	7.1 (2.4)	16.9 (6.3)	16.8 (5.0)
	June	Vindeln	S1	19.0 (0.5)	6.0 (0.4)	19.2 (1.0)	–
			S2	15.4 (0.8)	6.4 (0.6)	11.2 (0.3)	4.4 (0.4)
	July	Leuven	L6	13.8 (1.4)	6.9 (1.0)	16.9 (3.9)	16.3 (3.8)
	September	Leuven	L7	22.1 (3.7)	15.9 (3.2)	22.8 (6.0)	19.1 (5.9)

The crude associations of air pollution and blood pressure and carotid stiffness can be found in the online supplement (Additional file 1: Table S1) and the adjusted associations are presented in Table 3. After adjustment, arterial blood pressure parameters were not significantly associated with the studied pollutant concentrations. Nevertheless, we detected significant associations between the studied pollutant concentrations and the four measures of arterial stiffness. Five day average concentrations of PM_10_ and PM_2.5_ were significantly directly associated with PWV and YEM, and inversely with DC and CC. Five day average concentrations of NO_2_, both from monitoring stations and personal exposure samplers were inversely associated with CC.

**Table 3:** Adjusted^a^ changes (95% CI) in blood pressure and measures of arterial stiffness associated with an increase in five-day averages of PM_10_, PM_2.5_, or NO_2_ concentrations (n observations = 220)

	Monitoring stations			Personal exposure
	PM_10_	PM_2.5_	NO_2_	NO_2_
	per 10 μg/m^3^	per 5 μg/m^3^	per 10 μg/m^3^	per 10 μg/m^3^
Blood pressure (unit change)				
Systolic, mm Hg	−0.01 (−2.16;2.15)	0.26 (−0.72;1.24)	−0.98 (− 2.23;0.26)	−0.14 (−1.17;0.88)
Diastolic, mm Hg	− 1.14 (− 2.59;0.30)	− 0.28 (− 0.94;0.38)	−0.66 (− 1.52;0.19)	−0.28 (− 1.00;0.43)
Pulse pressure, mm Hg	1.11 (− 0.43;2.65)	0.53 (− 0.17;1.22)	−0.35 (− 1.24;0.54)	0.11 (− 0.62;0.83)
Carotid stiffness (% change)^b^				
PWV, m/s	**2.13 (0.80;3.47)**	**0.96 (0.32;1.59)**	0.78 (−0.05;1.61)	0.63 (− 0.04;1.30)
DC, 10^−3^/kPa	**−4.25 (−6.99;-1.51)**	**− 1.91 (− 3.21;-0.61)**	−1.58 (− 3.28;0.12)	−1.31 (− 2.69;0.07)
CC, mm^2^/kPa	**−4.65 (−7.05;-2.26)**	**−2.06 (− 3.20;-0.91)**	**−2.05 (− 3.54;-0.56)**	**−1.42 (− 2.64;-0.20)**
YEM, kPa	**4.18 (1.10;7.25)**	**2.07 (0.60;3.53)**	1.56 (− 0.34;3.45)	1.36 (− 0.14;2.86)

^a^Adjusted for age at baseline, sex, heart rate, smoking status, having a cold, medication use for blood pressure, date, temperature, relative humidity

^b^Models additionally adjusted for arterial pressure

Bold indicates *p*-value< 0.05

*PWV* Pulse wave velocity, *DC* Distensibility coefficient, *CC* Compliance coefficient, *YEM* Young’s elastic modulus

In sensitivity analyses excluding the trips to Milan and Vindeln (Additional file 1: Table S2 in the online supplement), PM_10_ and PM_2.5_ were statistically significant directly associated with ΔP. Regarding carotid stiffness, the exclusion of the trips resulted in additional statistically significant associations of NO_2_ measured from monitoring stations with PWV, DC and YEM in the hypothesized directions (i.e. direct for PWV and YEM and inverse for DC). However, the associations for personal exposure measures of NO_2_ lose statistical significance. Additional sensitivity analyses excluding individuals taking blood pressure medication or considering the effect of being a couple did not modify our results and the effect of couple was not statistically significant. Finally, interaction terms for sex and age were not statistically significant.

## Discussion

In a quasi-experimental study where we exposed 20 individuals to the range of ambient pollution levels that can be found in Europe, we found that changes in the vascular wall parameters of the carotid artery parallel exposure to five day average ambient air pollution (PM_10_, PM_2.5_, and NO_2_). This occurred without statistically significant associations with blood pressure measurements when including the trips to locations with extreme air pollution concentrations in Europe. Arterial stiffness and reduced elasticity were consistently associated with higher exposure to ambient air pollution. Young’s elastic modulus and pulse wave velocity, both direct measures of stiffness [28], were positively associated with PM, while the distensibility and compliance coefficient, both measures of elasticity [29], were negatively associated with five days exposure contrast to PM or NO_2_. This finding is in line with follow-up analyses of the Harvard Six Cities cohort study, showing a reduction in mortality risk in association with a decrease in ambient PM concentration [30, 31]. Our study adds evidence of the potential mechanisms involved in the associations observed between long and short term exposure to air pollution and cardio-vascular events.

Our results regarding arterial stiffness are in line with results from previous studies [17, 32–34]. Arterial stiffness is an important determinant of increased blood pressure and pulse pressure, and therefore a risk factor of events such as myocardial infarction and stroke [23, 35, 36]. Thus, our results provide a plausible biological mechanism for the demonstrated trigger effect of air pollution on myocardial infarction and stroke [1, 2, 5, 37]. However, the mechanisms responsible for the increase in stiffness by air pollution remain unknown. A proposed mechanism is an increase in inflammation and changes in cardiac autonomic function [9]. Also, diesel exhaust may enhance NO generation, altering the balance of basal generation and consumption of NO and resulting in low NO bioavailability, which would contribute to the cardiovascular effects of air pollution [38].

Regarding blood pressure, short-term or sub-acute (i.e. one week or less) exposure to high concentrations of air pollutants is associated with increases in systolic blood pressure and pulse pressure in healthy elderly, adults and children [39–43]. A recent panel study performed in Michigan (US) and Beijing (China) showed significant effects of sub-acute exposure to air pollution on blood pressure only in Beijing, suggesting that the effect of air pollution in normotensive healthy adults may only be evident in highly polluted areas [44]. In our study, we did not find statistically significant associations between blood pressure measurements and air pollution in the main analyses including all measurement time points. However, we did observe statistically significant direct associations for systolic blood pressure and pulse pressure when excluding the measurements performed during the trips. Therefore, it is possible that factors related with travelling have affected our results.

A 10-day group travel abroad is very different from the common home situation in many aspects and, although it allowed us to expose our volunteers to a wider range of pollutant concentrations, the trips themselves may also be considered as a limitation of our study. For example dietary changes such as switching to a “Mediterranean diet” in Milan could have attenuated effects of air pollution on blood pressure. In addition, it is possible that the stress of daily duties performed at home (e.g. taking care of grand children) disappeared during the trips. Other factors related to trips may include differences in alcohol consumption, or the amount of physical activity. Nevertheless, including the number of steps and alcohol consumption in our models or excluding the trips in the models for the measures of carotid stiffness did modify our results.

Another limitation of our study was the range of concentrations of air pollutants. We selected the study locations based on their annual PM averages. We expected to find ambient PM_10_ concentrations as low as 10 μg/m^3^ in rural Sweden and as high as 50 μg/m^3^ in Milan during several days in a row [12, 45]. However, PM concentrations during the study proved higher than expected in Vindeln (19.8 μg/m^3^) and lower than expected in Milan (30.6 μg/m^3^). Nevertheless, differences between locations were substantial for NO_2_. This may be explained by the fact that NO_2_ is more representative of traffic-related pollutants with larger spatial variation in ambient concentration than PM [46]. In addition, our 5-day average concentrations for data acquired from monitoring stations includes daily average concentrations on the day of the health measurements (lag0). This means that PM and NO_2_ data obtained from stations include pollution measured after the health measurement. Nevertheless, because we used 5-day average concentrations, the number of hours of pollutant measurements included after the health measurement is small and, therefore, unlikely to have biased our results. Moreover, the results for NO_2_ from monitoring stations are consistent with those obtained by personal sampling (which was stopped when starting the health measurement).

Nonetheless, our study counts on several strengths. It is a quasi-experimental longitudinal study with 11 measurement time points during one year and a relatively controlled exposure as compared with pure observational studies. During this period, we did not have any drop-out or important change in health status. Moreover, we used a large battery of objective health and exposure measurements, including personal exposure measures of NO_2_. The number of measurements included strongly increased the statistical power and the use of objective measurements reduced the potential for bias, allowing us to find subtle changes in cardiovascular health parameters related to air pollution.

## Conclusions

From a public health perspective, the findings presented here are relevant. Although the effects found on carotid arterial stiffness are small, previous studies reported that arterial stiffness predicts progression to hypertension in normotensive individuals [47–49]. Ambient air pollution is ubiquitous and the whole population is exposed, including more susceptible subgroups such as children, patients with pre-existing diseases, and elderly [50]. Consequently, small individual risks result in a large global burden [6]. Moreover, the time window of exposure in our study was relatively short, and people living in urban environments are continuously exposed to much higher levels of air pollution (http://www.who.int/phe/health_topics/outdoorair/databases/cities/en/). In our study, we found that decreases in air pollution exposure were associated with reduced arterial stiffness and improved elasticity. These observations demonstrate that measures leading to a reduction in exposure to air pollution are likely to have beneficial public health effects. In conclusion, our quasi-experimental study shows evidence for subacute effects of exposure to PM and NO_2_ on carotid stiffness. In this population age group, exposure to high concentrations of ambient pollutants within 5 days resulted in reduced elasticity of the common carotid artery.

## Additional file

Additional file 1: **Table S1.** Crude changes (95% CI) in blood pressure and measures of arterial stiffness associated with an increase in five-day averages of PM10, PM2.5, or NO2 concentrations (n observations = 220). **Table S2.** Adjusted changes (95% CI) in blood pressure and measures of arterial stiffness associated with an increase in five day average concentrations of PM10, PM2.5, or NO2 including only measurements performed in Leuven (Belgium) (n observations = 140). (DOCX 25 kb)

## Abbreviations

CC: Compliance coefficient
CI: 95% confidence interval
DALY: Disability-adjusted life years
DBP: Diastolic blood pressure
DC: Distensibility coefficient
PM: Particulate matter
PWV: Pulse wave velocity
SBP: Systolic blood pressure
YEM: Young elastic modulus
ΔP: Pulse pressure
