# Supplementary material for: Changing places to study short-term effects of air pollution on cardiovascular health: a panel study
Source: Environ Health. 2018 Nov 19;17:80. doi: 10.1186/s12940-018-0425-7 (PMC6245927; doi:10.1186/s12940-018-0425-7)
Supplement: Supplementary file 1 — Table S1. Crude changes (95% CI) in blood pressure and measures of arterial stiffness associated with an increase in five-day averages of PM10, PM2.5, or NO2 concentrations (n observations = 220). Table S2. Adjusted changes (95% CI) in blood pressure and measures of arterial stiffness associated with an increase in five day average concentrations of PM10, PM2.5, or NO2 including only measurements performed in Leuven (Belgium) (n observations = 140). (DOCX 25 kb) [file 12940_2018_425_MOESM1_ESM.docx]

**Supplementary material**

Table S1. Crude changes (95% CI) in blood pressure and measures of arterial stiffness associated with an increase in **five-day averages of** PM_10_, PM_2.5_, or NO_2_ **concentrations** (n observations=220).

|  |  | **Monitoring stations** | | |  | **Personal exposure** |
| --- | --- | --- | --- | --- | --- | --- |
|  |  | **PM_10_** | **PM_2.5_** | **NO_2_** |  | **NO_2_** |
|  |  | **per 10 µg/m³** | **per 5 µg/m³** | **per 10 µg/m³** |  | **per 10 µg/m³** |
| Blood pressure (unit change) | |  |  |  |  |  |
|  | Systolic, mm Hg | 0.58 (-1.10;2.26) | 0.65 (-0.12;1.42) | 0.36 (-0.57;1.29) |  | **0.62 (0.00;1.23)** |
|  | Diastolic, mm Hg | -0.27 (-1.44;0.91) | 0.19 (-0.34;0.72) | 0.28 (-0.40;0.97) |  | 0.44 (-0.08;0.95) |
|  | Pulse pressure, mm Hg | 0.86 (-0.45;2.17) | 0.46 (-0.07;0.99) | 0.09 (-0.56;0.73) |  | 0.18 (-0.25;0.60) |
| Carotid stifness (% change) | |  |  |  |  |  |
|  | PWV, m/s | **2.04 (1.02;3.06)** | **0.88 (0.38;1.38)** | **0.71 (0.11;1.31)** |  | **0.46 (0.06;0.86)** |
|  | DC , 10^−3^/kPa | **-4.12 (-6.21;-2.02)** | **-1.77 (-2.78;-0.76)** | **-1.45 (-2.68;-0.21)** |  | **-0.95 (-1.77;-0.13)** |
|  | CC, mm^2^/kPa | **-4.69 (-6.52;-2.85)** | **-1.99 (-2.87;-1.10)** | **-1.88 (-2.97;-0.79)** |  | -5.98 (-12.31;0.35) |
|  | YEM, kPa | **3.82 (1.53;6.11)** | **1.91 (0.81;3.00)** | **1.46 (0.15;2.78)** |  | **0.97 (0.09;1.85)** |

Bold indicates p-value<0.05. Lag0: average concentrations on the day of the health measurement; PWV: pulse wave velocity; DC: distensibility coefficient; CC: compliance coefficient; YEM: Young’s elastic modulus;

Table S2. Adjusted^a^ changes (95% CI) in blood pressure and measures of arterial stiffness associated with an increase in five day average concentrations of PM_10_, PM_2.5_, or NO_2_ including only measurements performed in Leuven (Belgium) (n observations=140).

|  |  | **Monitoring stations** | | |  | **Personal exposure** |
| --- | --- | --- | --- | --- | --- | --- |
|  |  | **PM_10_** | **PM_2.5_** | **NO_2_** |  | **NO_2_** |
|  |  | **per 10 µg/m³** | **per 5 µg/m³** | **per 10 µg/m³** |  | **per 10 µg/m³** |
| Blood pressure (unit change) | |  |  |  |  |  |
|  | Systolic, mm Hg | 2.37 (-0.94;5.69) | 0.93 (-0.74;2.61) | -0.35 (-3.72;3.01) |  | -0.05 (-3.81;3.71) |
|  | Diastolic, mm Hg | -0.50 (-2.59;1.59) | -0.38 (-1.43;0.67) | 0.00 (0.05;-2.72) |  | -0.63 (-3.18;1.92) |
|  | Pulse pressure, mm Hg | **3.36 (0.70;6.02)** | **1.47 (0.21;2.73)** | 0.25 (-2.08;2.57) |  | 0.46 (-1.98;2.90) |
| Carotid stifness (% change)^b^ | |  |  |  |  |  |
|  | PWV, m/s | **3.89 (1.63;6.16)** | **1.72 (0.57;2.88)** | **2.58 (0.24;4.91)** |  | 1.14 (-1.43;3.70) |
|  | DC , 10^−3^/kPa | **-7.78 (-12.31;-3.25)** | **-3.44 (-5.76;-1.13)** | **-5.16 (-9.83;-0.48)** |  | -2.27 (-7.40;2.86) |
|  | CC, mm^2^/kPa | **-7.15 (-12.55;-1.74)** | **-3.01 (-5.76;-0.26)** | -4.85 (-10.30;0.61) |  | -2.47 (-8.61;3.67) |
|  | YEM, kPa | **8.47 (3.81;13.12)** | **4.12 (1.79;6.46)** | **5.78 (1.22;10.33)** |  | -0.63 (-6.11;4.84) |

^a^Adjusted for age at baseline, sex, heart rate, smoking status, having a cold, medication use for blood pressure, date, temperature, relative humidity.

^b^Models additionally adjusted for arterial pressure.

Bold indicates p-value<0.05. Lag0: average concentrations on the day of the health measurement; PWV: pulse wave velocity; DC: distensibility coefficient; CC: compliance coefficient; YEM: Young’s elastic modulus;
